# Leaky gut in systemic inflammation: exploring the link between gastrointestinal disorders and age-related diseases

**DOI:** 10.1007/s11357-024-01451-2

**Published:** 2024-12-06

**Authors:** Jonathan Escalante, Olivia Artaiz, Shanti Diwakarla, Rachel M. McQuade

**Affiliations:** 1 Gut-Barrier and Disease Laboratory, Department of Anatomy and Physiology, The University of Melbourne, Melbourne, VIC 3021 Australia; 2 The Florey Institute of Neuroscience and Mental Health, Parkville, VIC 3010 Australia; 3 Australian Institute for Musculoskeletal Science (AIMSS), The Melbourne University and Western Health, Melbourne, VIC 3021 Australia

**Keywords:** Aging, Inflammation, Inflammageing, Leaky gut, Systemic inflammation

## Abstract

Global average life expectancy has steadily increased over the last several decades and is projected to reach ~ 77 years by 2050. As it stands, the number of people > 60 years currently outnumbers children younger than 5 years, and by 2050, it is anticipated that the global population of people aged > 60 years will double, surpassing 2.1 billion. This demographic shift in our population is expected to have substantial consequences on health services globally due to the disease burden associated with aging. Osteoarthritis, chronic obstructive pulmonary disease, diabetes, cardiovascular disease, and cognitive decline associated with dementia are among the most common age-related diseases and contribute significantly to morbidity and mortality in the aged population. Many of these age-related diseases have been linked to chronic low-grade systemic inflammation which often accompanies aging. Gastrointestinal barrier dysfunction, also known as “leaky gut,” has been shown to contribute to systemic inflammation in several diseases including inflammatory bowel disease and irritable bowel syndrome, but its role in the development and/or progression of chronic low-grade systemic inflammation during aging is unclear. This review outlines current literature on the leaky gut in aging, how leaky gut might contribute to systemic inflammation, and the links between gastrointestinal inflammatory diseases and common age-related diseases to provide insight into a potential relationship between the intestinal barrier and inflammation.

## Introduction

Global life expectancy has increased thanks to advancements in medicine and improvements in living standards. By 2025, it is expected that 1.2 billion people will be over the age of 60, and by 2050, this number will increase to 2 billion [3]. Although this is generally viewed as a positive outcome, the unfortunate fact is that the continuous process of aging comes with a progressive decline in overall organ and systemic function, in particular, the immune system [19]. This age-related dysregulation of the immune system is termed immunosenescence. Immunosenescence refers to a gradual deterioration of the immune system associated with aging that impacts both innate and acquired immunity [3, 19, 37, 38]. A hallmark of immunosenescence is the replacement of naïve T and B cells with memory cells, which occurs due to age-related atrophy of the bone marrow and thymus, and this decline in lymphopoiesis leads to diminished adaptive immune responses. In parallel, the functionality of mature lymphocytes in secondary lymphoid tissues is impaired, further weakening immune responses to novel antigens [38, 56, 109]. However, while some aspects of immunity do indeed deteriorate with age, some become overactive and others tend to remain unchanged [112]. This forms the premise for the contrary concept of age-related immune remodeling which reflects a shift in immune profiles rather than just a decline. The premise of age-related immune remodeling centers around the thought that decades of exposure to infections, medications, and various other environmental stimuli shape the aging immune system, impairing some functions, improving others, and having no effect on others [112]. However, progressive increases in the incidence of cancer, neurological disorders and infectious, metabolic, and autoimmune conditions with aging appear likely to be at least partly caused by immunosenescence, impacting the body’s ability to respond to infection, leaving aged individuals more susceptible to pathogens and inflammatory processes that can trigger disease [104, 105, 112, 113].

Simultaneously, a multitude of factors converges to promote a state of chronic low-grade inflammation, a process dubbed “inflammaging.” Inflammaging is influenced by several factors that interrelate with immunosenescence and/or age-related immune remodeling. These include the accumulation of senescent cells that secrete pro-inflammatory cytokines, an imbalance between pro- and anti-inflammatory signals, and reduced clearance of cellular debris. The immune system’s dysregulation contributes to a feed-forward loop, exacerbating systemic inflammation while impairing the ability to resolve it.

Inflammaging has been linked to many age-related diseases such as cardiovascular disease (CVD), type-2 diabetes (T2D), chronic obstructive pulmonary disease (COPD), cognitive decline associated with dementia, and osteoarthritis (OA) [10, 19, 38, 56]. While the cellular changes that occur in the immune system with aging have been heavily studied over the last several decades, only recently has the gastrointestinal (GI) tract emerged as a potential contributor to age-related systemic inflammation. The GI tract is one of the most sophisticated organs in our body and functions to secrete, digest, and absorb nutrients while simultaneously preventing pathogens, toxins, and other harmful substances from entering the body. The structural, molecular, and microbial components that collectively constitute the intestinal barrier, including epithelial cells, tight junction proteins, adhesion molecules, mucous membrane, and resident immune cells, which all play an important role in fulfilling the complex, but essential function of the GI tract [165]. Thus, disruption at any level of the intestinal barrier can have significant systemic consequences.

Recent work has found that aging is associated with degradation of the intestinal barrier, hyperpermeability, and inflammation [137, 162], all of which are hallmarks of “leaky gut.” Changes in intestinal permeability are believed to promote the passage of bacteria and harmful molecules through the mucosal barrier and into systemic circulation, inducing an inflammatory response [120, 162]. Leaky gut is known to contribute to systemic inflammation in several diseases including inflammatory bowel disease (IBD), irritable bowel syndrome (IBS), and several autoimmune conditions, but its role in the development and/or progression of chronic low-grade systemic inflammation in human aging is unknown.

Here, we review the current literature surrounding intestinal barrier dysfunction and leaky gut in aging, how this may contribute to the development and progression of systemic inflammation, and explore the links between leaky gut and the most common age-related diseases.

## The Gastrointestinal Tract

The GI tract, or gut, refers to the succession of organs stretching from the mouth to the anus. It consists of the esophagus, stomach, small intestine, and large intestine [2] and represents the largest mucosal surface in the body. The GI barrier, which lines the length of the GI tract and directly interfaces with external environmental factors, plays an important role in shielding the body from noxious environmental influences while simultaneously preserving the ability to absorb nutrients [18]. The intestinal barrier is not static but rather a complex multilayer system consisting of physical (mucus, epithelial cells, tight junctions), biochemical (bile salts, enzymes, antibacterial proteins), immunological (IgA and immune cells), and microbial components that interact to selectively absorb nutrients while simultaneously preventing the infiltration of pathogenic molecules [27]. The integrity of this dynamic barrier is maintained via bidirectional communication between specialized epithelial cells, immune cells, intrinsic and extrinsic nerve cells, and the microbiome and relies heavily on the regulation and function of several key tight junction protein complexes including claudin and occludin [165]. Tight junctions are crucial structures located at the apical region of epithelial cells that line the intestinal barrier. These protein complexes form a seal between adjacent cells, regulating the paracellular transport of ions, water, and small molecules [31]. In the intestines, tight junctions are essential for maintaining the selective permeability that allows nutrients to pass into the bloodstream while keeping harmful pathogens, toxins, and antigens out. Claudins are a family of proteins which, along with occludin, are the most important components of the tight junction protein complex. Claudins play a critical role in determining the permeability properties of tight junctions, allowing the selective passage of specific ions and molecules [31]. Occludin, by contrast, contributes to the stability and maintenance of tight junctions, as well as signalling processes that regulate barrier function and cellular behavior [29].

Alteration to either the physical or functional components of the intestinal barrier has been shown to contribute to GI dysfunction, malnutrition, local inflammation, and systemic inflammation [165].

## Intestinal barrier dysfunction *AKA* “leaky gut” in inflammatory conditions

Broadly speaking, leaky gut refers to a state of intestinal hyperpermeability, which, in the most well-characterized conditions is accompanied by intestinal inflammation and morphological/structural changes in the epithelial layer of the intestine [24]. Leaky gut has been described in a vast array of conditions, including non-alcoholic steatohepatitis (NASH), dementia [92, 152], autism [42, 50], anxiety/depression [102, 153], and chronic heart failure [55]. However, by far, the most widely studied and well-characterized conditions associated with leaky gut are the GI inflammatory disorders IBD and IBS. Drawing parallels between GI disorders, where intestinal permeability and systemic inflammation are prominent features, and common age-related diseases may provide insights into the potential role of the intestinal barrier in systemic inflammation in aging. This will be further discussed in Sect. 7.0.

Cumulative research over the last several decades has shown that IBD patients display increased intestinal permeability [147, 177], which has been linked to abnormalities in tight junction proteins, including decreased expression and distribution of occludin, claudins, and junctional adhesion molecules [47, 88, 93, 138, 166, 189]. A reduced number of goblet cells [61], thinning of the mucus layer [130], and an altered mucus composition have also been found in individuals with IBD and ulcerative colitis (UC) [91, 164]. In addition, longitudinal studies in patients with IBD suggest that increased intestinal permeability precedes Crohn’s disease (CD) [40] hinting at a potential role of the intestinal barrier in the pathogenesis of gut inflammation.

Similarly, evidence in IBS patients indicates that there are distinct pathological changes in the intestinal barrier. Small intestinal permeability has been found to be significantly increased in individuals with IBS-diarrhea when compared to healthy controls [111] and reduced expression of tight junction proteins, namely zonula occludens (ZO)−1 and E-cadherin, have been observed in the colon of patients with IBS [15, 33, 178]. More recently, patients with postinfectious (PI)-IBS showed significantly higher serum intestinal fatty acid binding protein I than healthy controls, an indirect marker of intestinal permeability reflecting the integrity of the intestinal barrier, indicating intestinal injury and/or intestinal inflammation [143]. In support of this, several studies have shown low-grade immune activation and increased infiltration of the colonic mucosa by several immune cells including T lymphocytes and mast cells in IBS [26, 45, 126, 149]. Changes in the intrinsic enteric nervous system, including lymphocytic infiltration and neuronal degeneration have also been noted in full-thickness jejunal specimens from severe IBS patients [158].

Studies have also shown that the gut microbiome is also altered in patients with IBD compared with healthy control subjects [62]. In both CD and UC patients, there is decreased biodiversity, a lower proportion of *Firmicutes*, and an increase in *Gammaproteobacteria* [148]. Consistent changes in the gut microbiome in individuals with IBD include an increase in facultative anaerobes, including *Escherichia coli* [85], alongside a decrease in anaerobic producers of short-chain fatty acids [110]. In IBS, a growing number of studies demonstrate that the diversity, stability, and metabolic activity of the gut microbiome are altered in IBS patients compared with healthy individuals. Diversity of fecal microbiota has been found to be consistently reduced in IBS patients [73, 128], and a recent systematic review uncovered that family *Enterobacteriaceae*, family *Lactobacillaceae*, and genus *Bacteroides* were increased in patients with IBS compared to controls, whereas genus *Faecalibacterium* and genus *Bifidobacterium* were decreased [128]. Importantly, a recent correlation between microbiota and clinical manifestations in IBS patients showed that *Bacteroides caccae* and *Roseburia* in fecal samples and *Bifidobacterium* and *Eubacterium* in intestinal mucosal samples were associated with abdominal pain and distention compared with healthy controls [73].

While the term “leaky gut” is widely utilized among the general public and academics alike, it remains to be recognized as a clinical condition. This is, in no small part, due to the limitations of current diagnostic tools and the complexities that accompany the interpretation of their results [24, 132]. Consequently, pathological hallmarks and diagnostic criteria for leaky gut remain undefined, and intense conflict surrounds whether or not the term “leaky gut” can, and should, be used outside the realm of GI inflammatory disorders [24].

## Changes in the aging gut

Aging has been associated with alterations in various components of the intestinal barrier in animals and humans alike. Age-associated remodeling of the intestinal epithelium, altered expression of tight junction proteins, inflammation, and changes in enteric neuron density, particularly in the myenteric plexus has been reported.

## Structural changes in the aging gut

Only a handful of studies have investigated the effects of aging on intestinal structure and morphology in human tissue [34, 96, 106, 160, 174, 175] and have yielded contradictory findings (Table 1). Early studies assessing structural changes in intestinal morphology in humans uncovered differences between the proximal jejunal mucosa of elderly subjects (67–90 years) and younger controls (13–59 years) [175] (Table 1). The study reported that “leaf-shaped” villi were more commonly seen among older vs. younger subjects, and average villous height among elderly subjects was significantly smaller than pooled results of previous publications in young subjects [175] (Table 1). Similarly, a follow-up study examining upper jejunal biopsy sections from 10 (well-nourished) elderly patients (aged 60–73) and 10 specimens from younger patients (aged 16–30) found there was a highly significant reduction in mucosal surface area in elderly patients [174] (Table 1). A more recent study utilizing computer-aided morphometric analysis comprehensively assessed jejunal and ileal mucosa in adult (< 60 years) and elderly (> 60 years) subjects and found significant age and sex-associated changes [160] (Table 1). Jejunal mucosal thickness was significantly reduced in elderly subjects, especially in elderly females compared to adult ones, and jejunal villi were significantly wider in adults than in the elderly subjects, while ileal villi were significantly wider in elderly compared to adult subjects and in males compared to female subjects [160] (Table 1). The same group also conducted computer-aided morphometric analysis in the rectal mucosa of adult and aged individuals. While a significant decrease in the height of surface epithelium was detected, the changes associated with aging overall were discrete and appeared to affect only the male subjects [106] (Table 1).

**Table 1:** Summary of intestinal histological and morphometric findings

Authors	GI region	Age range	Result
Webster and Leeming [175]	Proximal jejunum biopsy/specimens	< 60 vs. > 60 Mean age controls = 43 Mean age aged = 80	↓ Villous height
Warren et al. [174]	Upper jejunal biopsy	< 30 vs. > 60 Controls = 16–30 Aged = 60–73	↓ Mucosal surface area
Corazza et al. [34]	Jejunal biopsy	Young subjects = 22 Elderly subjects = 16	↔ Surface area to volume ratio of mucosa, mean enterocyte height
Lipski et al. [96]	Duodenal biopsy	< 70 vs. > 70 Median age across cohort = 69	↔ Duodenal surface epithelium, height/width villi, depth/width crypt, crypt to villus ratio
Milosevic et al.[106]	Rectal biopsy	< 60 vs. > 60 Control male = 42 Control female = 50 Aged male = 72 Aged female = 72	↓ Mucosal surface area
Trbojević-Stanković et al. [160]	Jejunal and ileal biopsy	< 60 vs. > 60 Mean age range controls 42–49 mean age range aged 70–77	↓ Jejunal mucosal thickness ↓ Jejunal villi width ↑ Ileum villi width

While these studies point to potential age-related structural differences in the upper small intestines, a small collection of studies assessing duodenal and jejunal morphology have found no difference in intestinal morphology when comparing young and aged individuals. Corazza and colleagues found no significant difference in the surface-to-volume ratio of jejunal mucosa or enterocyte height when comparing jejunal biopsies from 22 young and 16 elderly subjects [34] (Table 1). Similarly, a study investigating small bowel morphometry in 25 subjects < 70 years and 22 subjects > 70 years found no significant correlations between age and area of duodenal surface epithelium, area of crypts, area of lamina propria, heights of surface epithelium and villi, crypt depth, crypt to villus ratio, or the number of intraepithelial lymphocytes [96] (Table 1. Interestingly, the authors did find that villus height and crypt depth were slightly reduced in > 70 subjects; however, the difference did not reach significance.

The divergence in morphological and structural findings from human intestines over the last 4 decades and whether or not they contribute to malabsorption and/or malnutrition in the elderly has been heavily debated. Difficulty in defining and obtaining “normal” biopsy controls from healthy individuals and variations in exclusion and selection criteria across studies have complicated interpretations and are likely factors contributing to discrepancies in reported data. While some studies select “healthy” controls based on general health, a subset has extensive exclusion criteria, and others choose subjects based on the structural normality of the mucosa. The nutritional status of subjects is another confounding factor, some studies purposely excluded subjects with evidence of malabsorption [96, 174], others specifically selected aged individuals based on compromised absorption of nutrients [175], and the remaining studies did not appear to assess nutritional status [106, 160].

While most studies were conducted in the upper small intestine, regional differences across the duodenum, jejunum, and ileum as well as variation in regional boundaries and site of tissue collection across studies may explain the wide range of values reported for villi length and width. Finally, differences in age bracket distinction and sex of participants could contribute to deviation across reported findings.

As the absorption of many substances is dependent on mucosal surface area availability, the above-described changes in aging individuals could account for the small bowel functional impairment suspected by many geriatricians and gerontologists [175].

## Mucosal and microbial changes in the aging gut

Throughout the GI tract, the role of mucins and the mucosal layer is two-fold; acting to slough away unwanted bacteria and providing a physical barrier between the epithelium and the lumen of the GI tract. Despite its important role, all but three studies have investigated the effects of aging on intestinal mucus in humans, yielding conflicting results.

An early study by Vliegenthart and colleagues found that the total sialic acid concentration in human gastric aspirates decreased with age, suggesting a structural change in gastric mucus [167]. A follow-up study investigating parietal and mucus cell mass in the gastric mucosa of adults ranging from 22 to 65 years of age reported a reduction in mucous cells in the fundic mucosa with age [48]. Later work directly assessing gastric antral mucus thickness and duodenal mucus thickness via endoscopy found that in healthy (*H. pylori negative*) subjects, there was no correlation between mean mucus thickness, in either the stomach or duodenum, and age [116].

Given the important role of intestinal mucus in both the physical protection of the intestinal barrier and digestive processes, further studies are required to determine whether mucus production and/or secretion has any impact on intestinal barrier function during aging.

The relationship between the intestinal mucous layer and microbiome is well-established, and indeed, several studies have reported a potential involvement of mucin-degrading bacteria in the pathogenesis of altered gut microbiome AKA “microbial dysbiosis” and intestinal diseases. Furthermore, compositional differences in specific bacterial species have been identified in the elderly human gut microbiome compared to the young adult microbiome (Table 2).

**Table 2:** Summary of microbial changes in key aging and longevity studies

Authors	Study details	Microbial changes
He et al. [67]	fluorescent in situ hybridization (FISH) and 16sRNAseq 15 elderly subjects > 75 years and an unknown number of healthy volunteers (aged 20–55 years)	↑ Abundance of *Ruminococcus*, *Eubacterium*, *Lactobacillus*, and *Enterococcus* ↓ Abundance of *Faecalibacterium* and *Bacteroides*
Yatsunenko et al. [187]	16sRNAseq,	↓ Abundance *Bifidobacterium Longum*
Biagia et al. [17]	16sRNAseq 69 subjects from Emilia Romagna, Italy 22–109 years	↑ Abundance of *Oscillospira*, *Odoribacter*, and *Butyricimonas* ↓ Abundance of *Coprococcus, Roseburia*, and *Faecalibacterium*
Kong et al. [87]	16sRNAseq 168 subjects from Dujiangyan and Ya’an, Sichuan province, China 24–102 years	↑ Abundance of *Lachnospiraceae*, *Ruminococcaceae*, and *Erysipelotrichaceae* *Blautia*, *Faecalibacterium*, *Escherichia_Shigella*, and *Clostridium XIVa* cluster
Odamaki et al. [118]	16sRNAseq 367 healthy Japanese subjects 1–104 years	↓ Abundance of *Bacteroidetes*, *Betaproteobacteria*, and *Deltaproteobacteria*
*Badal et al.[ 9]	Systematic review of 27 papers	↑ Abundance *Akkermansia* ↓ Abundance of *Faecalibacterium*, *Bacteroidaceae*, and *Lachnospiraceae*

With regard to alpha diversity, that is the diversity applicable to a single sample, studies have yielded inconsistent and opposing findings. While a vast majority of studies have reported increasing alpha diversity with aging/higher levels of alpha diversity in the long-living groups [87, 118, 161, 185, 187], several studies have found no differences in alpha diversity when comparing young adult and old subject [83, 181].

Early works conducted by He and colleagues reported an age-related upregulation of *Ruminococcus*, *Eubacterium*, *Lactobacillus*, and *Enterococcus* in elderly (> 75 years) subjects when compared to an adult cohort (20–55 years), as well as a reduction in *Faecalibacterium* and *Bacteroides*, suggesting a potential shift away from “health-promoting” bacteria in aging [67]. In agreement with this, years later a seminal paper by Yatsunenko and colleagues investigating gut microbiota composition across the lifespan in three distinct geographical locations (Amazonas of Venezuela, rural Malawian residents, and inhabitants of the US metropolitan areas) found that *Bifidobacterium longum* significantly decreased with increasing age in all three populations [187] (Table 2). Similarly, a cross-sectional study investigating age-related changes in microbiota from infancy to 104 years of age in a Japanese population revealed distinct patterns and transition points in the composition of fecal microbiota during aging [118]. When subjects were divided into three age clusters (infant, adult, and elderly), certain transition types of microbiota were found to be enriched in infants, adults, and elderly individuals [118]. The transition from infancy to the elderly was accompanied by a distinctive co-abundance group dominance with *Bacteroides*, *Eubacterium*, and *Clostridiaceae* (Table 2). The elderly cluster showed a significantly higher abundance of *Bacteroidetes*, *Betaproteobacteria*, and *Deltaproteobacteria* (Table 2). Interestingly, certain oral bacteria, which often have difficulty reaching the GI tract due to the presence of gastric juice and bile acid, such as *Porphyromonas*, *Treponema*, *Fusobacterium*, and *Pseudoramibacter* were also found to be enriched in the elderly associated co-abundance groups [118].

In a population of Italian subjects including 24 semi-supercentenarians (> 105 years of age), Biagi and colleagues revealed that the abundance of *Coprococcus*, *Roseburia*, and *Faecalibacterium*, of the *Lachnospiraceae* and *Ruminococcaceae* families, were negatively associated with age, while *Oscillospira* and two subdominant members of the *Bacteroidales* order (*Odoribacter* and *Butyricimonas*) were positively correlated with age [17] (Table 2).

A follow-up study by Kong and colleagues aiming to identify microbial signatures that best differentiate between the long-living (> 90 years), elderly (65–83 years), and young (24–64 years) individuals across a Chinese population and data derived from Biagi et al. [17] found that despite differences in the overall community structures, common features could be identified in both groups that discriminate long-living from young people [87]. Among the top 50 features that differentiate the Chinese long-living people from the younger groups, 11 were also listed as the top 50 in the Italian dataset by Biagi and colleagues. Enrichment of families *Lachnospiraceae*, *Ruminococcaceae*, and *Erysipelotrichaceae*, *Faecalibacterium*, *Escherichia_Shigella* genus, and *Clostridium* XIVa cluster was found in the long-living groups in both cohorts [87] (Table 2).

A recent systematic review including 27 papers focused on microbial composition in the context of aging and longevity concluded that overall *Akkermansia* was most consistently reported to be relatively more abundant with aging, whereas *Faecalibacterium*, *Bacteroidaceae*, and *Lachnospiraceae* were relatively reduced [9] (Table 2).

How alterations in these specific species could impact intestinal barrier integrity, and permeability is multifaceted. An increasing number of experimental studies have demonstrated the protective effects of *Akkermansia* in the intestine [107]. In mice, *Akkermansia muciniphila* has been found to stimulate the production of mucus by goblet cells [84], which could reduce the risk of barrier dysfunction. It has also been shown to positively influence the expression of tight junction proteins, such as occludin and claudins, through AMP-activated protein kinase activation to decrease intestinal permeability of lipopolysaccharide [6, 28], thereby supporting intestinal barrier integrity. Further to this, *Akkermansia* has also been associated with anti-inflammatory effects in the gut, through the expansion of regulatory T cells, and the production of short-chain fatty acids, such as acetate and propionate, which have been shown to directly modulate the epithelial immune response and enhance intestinal barrier function [145].

Reductions in *Faecalibacterium*, *Bacteroidaceae*, and *Lachnospiraceae* have been linked to pro-inflammatory status and altered expression of tight junction expression in the intestine [81, 101]. *Faecalibacterium prausnitzii* is known for its anti-inflammatory properties and has been linked to improved gut barrier function due to its role in butyrate production. Butyrate has been shown to enhance tight junction integrity by upregulating the expression of key tight junction proteins, including claudins-1,3 and 4 [186]. Reduction in *Faecalibacterium* may also lead to a decrease in its anti-inflammatory effects, such as the suppression of pro-inflammatory cytokines (e.g., IL-6, IL-1β). Like *Faecalibacterium*, some members of the *Lachnospiraceae* are significant producers of butyrate [57], which could have downstream effects on tight junction protein expression. Butyrate can also stimulate dendritic cells and regulatory T cells to increase IL-10 secretion in the intestine [13], indirectly influencing the intestinal immune environment. Further to this, it has been shown that *Lachnospiraceae* can directly interact with intestinal epithelial cells to promote cytokine production [134].

Certain members of the *Bacteroidaceae* family, such as *Bacteroides*, can have dual roles at the intestinal barrier, and their activity can become pro-inflammatory under specific conditions. *Bacteroidaceae* species are known mucin degraders, which are generally beneficial for turnover in the mucus layer [100]. However, if the activity of mucin-degrading *Bacteroides* becomes imbalanced, the mucus layer may thicken or thin excessively. This can compromise the mucus layer allowing for pathogenic bacteria, toxins, and inflammatory molecules to come into direct contact with the epithelial cells, triggering an inflammatory response and damaging the intestinal barrier [129].

Though there is expanding knowledge on the role of specific microbial species in promoting pro and anti-inflammatory immune status in a variety of health conditions, it is worth noting that strain level variation is important, and not every member of a family has the same abilities. Our understanding of how microbial dysbiosis might contribute to or mediate both leaky gut and inflammation with advancing age is incomplete. Studies conducted in mice have highlighted that microbial dysbiosis can alter intestinal permeability, contributing to systemic inflammation [156] and that “aged” microbiota can trigger an exaggerated systemic inflammatory response when transferred into young mice, potentially through microbial translocation, proposing a causative role for microbiota in age-associated chronic low-grade systemic inflammation [53, 151, 156]. Microbial translocation refers to the phenomenon whereby bacterial components such as lipopolysaccharides (LPS), peptidoglycans, and flagellins bypass the epithelial cell layer and enter systemic circulation [22]. Once bacterial components enter the bloodstream, they can be recognized by the innate immune system, particularly monocytes, macrophages, and dendritic cells. These cells express pattern recognition receptors (PRRs) that detect microbial-associated molecular patterns (MAMPs). Upon recognition by PRRs, signaling pathways such as NF-κB and MAPK are activated, leading to the transcription of several pro-inflammatory cytokines including TNF-α, IL-6, IL-1β, and IFN-γ [140].

Similarly, correlative data from human studies have demonstrated that bacteria of the phylum *Proteobacteria* are positively correlated with IL-6 and IL-8, while *Ruminococcus lactaris* is negatively correlated with IL-8 [144].

Cumulatively, these data suggest that aging-related shifts in microbial composition may be a contributing factor to inflammatory responses that occur with advancing age [118].

## Neuronal changes in the aging gut

A reduction in the number of enteric neurons during aging has been described in most, but not in all studies [137]. To date, aging has been associated most heavily with a decrease in the total number and density of enteric nerve fibers, particularly in the myenteric plexus of the colon [11, 12, 43, 63, 66].

Early work conducted by De Souza and colleagues investigating the density of myenteric neurons in the duodenum, jejunum, and ileum in autopsy material of six young (average age of 32) and six old individuals (average age of 71) found a significant reduction in the number of neurons in the ganglia of the myenteric plexus of the old subjects in all regions of the small intestine [43], with the largest proportional loss (38%) seen in the duodenum. A follow-up study conducted by the same group assessing myenteric neuron density in segments of ascending, transverse, descending, and sigmoid colon from six young (average age of 30) and six old subjects (average age of 77) also found a significant reduction in myenteric neurons density between young and old individuals [63]. The average density of myenteric neurons across the colon decreased by approximately 37% in the aged cohort, while collagen and elastic system fibers were more numerous. Interestingly, though Gomes and colleagues found no significant difference in neuronal cell body size when comparing young and old individuals [63], later studies examining colonic samples from 168 patients aged 10 days to 91 years found age-related changes in the size and appearance of myenteric ganglia [66]. In samples from older individuals, the overall ganglionic area was found to be larger, and gaps or spaces were observed within the ganglia, particularly in the colon, where the proportion of ganglia with cavities was 2.56 times greater than in the ileum [66]. In agreement with this, a more recent study has also confirmed that the number of Hu-positive neurons in the myenteric plexus of the colon declines with age [12].

Age-related change in the chemical phenotype of myenteric neurons has also been noted in several studies. Early work by Belai and Bernstock (1999) found a 26% increase in the proportion of nicotinamide adenine dinucleotide phosphate (NADPH)-diaphorase-positive (labels NOS neurons) and a 21% increase in calretinin-positive neurons in the myenteric plexus of the small intestine in aged individuals (average age of 81 years) compared to adults (average age of 55 years) [11]. Similarly, while the number of Hu-positive and choline acetyltransferase (ChAT)-positive neurons in the myenteric plexus of the colon has been found to decline with age, the total number of neuronal nitric oxide synthase (nNOS)-positive neurons remained unchanged, resulting in a proportional increase of nNOS to Hu within the myenteric plexus [12]. Recent evidence also suggests impaired myenteric neuromuscular function in aged colonic samples [23, 193].

Far fewer studies have investigated changes in the number of neurons in the submucosal plexus during aging in humans. A single study has found that while the number of neurons, neurons per ganglion, number of ChAT-positive or number of nNOS-positive cells did not change with age, and the average number of ganglions per area/length decreased with age [12].

Changes in the density, structure, and neurochemical characteristics of enteric neurons have previously been linked to gastrointestinal dysfunction in various conditions, such as IBD, IBS, Hirschsprung’s disease, and achalasia [1]. There has been a growing recognition of the relationship between age-related ENS changes and gastrointestinal symptoms in aging [51].

Gastrointestinal dysfunction is prevalent in the aged population [137]. Disorders such as dysphagia, reflux, chronic constipation, fecal impaction, and incontinence are frequently observed, while delayed gastric emptying and impaired absorption, which may be associated with bacterial overgrowth, have been noted in certain studies [51]. Chronic constipation is one of the most common complaints among older patients [36]. In the wider community, around 15% of adults experience chronic constipation, this figure rises to 30–40% in those aged over 65 [58]. Over 50% of adults residing in care facilities suffer from chronic constipation, with nearly 74% of this group relying on daily laxatives.

While enteric neuropathy may be a contributing factor in gastrointestinal dysfunction, it must be noted that age-related constipation could arise from various factors, including insufficient fluid intake, inadequate nutrition, adverse effects from medications, and both acute and chronic health conditions, such as the aftermath of a stroke. Furthermore, investigations of colonic function in aging should be interpreted cautiously given the incidence of long-term/chronic laxative use which has been linked to loss of interneurons, specifically phenolphthalein.

## Intestinal inflammation in aging

The GI tract is considered one of the largest immunological organs in the body, harboring an estimated 70% of the body’s lymphocyte population, and consequently plays a central role in regulating global immune homeostasis [155]. It has been suggested that age-associated alterations that arise in the mucosal immune system of the GI tract occur earlier than in other systemic immune compartments [86]. These changes may impair the protective mechanisms of the intestinal barrier and thus contribute to intestinal barrier dysfunction [116].

There is evidence of increased intestinal inflammation in aging [141]. A detailed study investigating the dynamics of intestinal immune parameters over the human life span recently assessed lymphocyte localization in the ileum, jejunum, and colon of individuals aged from 4 months to 87 years (*n* = 68). Subjects were classified into three life stages: young (0–24 years), middle (25–49 years), and older (50 + years), with results showing age-related changes in distribution and lymphocyte composition [141]. Isolated lymphoid follicles in the jejunum of the young population were found to be increased in density and relative size compared to those of individuals in the middle and older populations, although similar numbers and density were maintained in the colon in all three age groups. Furthermore, the CD4:CD8 T cell ratio was found to be reduced in Peyer’s patches of middle and older populations when compared to young, in both small and large intestines [141]. Taken together, the reduced lymphoid density and CD4:CD8 T cell ratio could suggest altered immune capacity with age [60]. However, it must be noted that while age-related changes in CD4:CD8 T cell ratios are well defined in mice and other animal models [7, 103, 115, 127], they are harder to link to a functional consequence in humans.

Similar work conducted in rhesus macaques confirmed that aging was associated with a decline in CD161 + cells and reduced expression of IL-22 and IL-17, cytokines that protect against intestinal hyperpermeability, and higher plasma levels of inflammatory cytokines IL-6, TNF-α, IL-1β, GM-CSF, IL-12, and Eotaxin [170]. These changes were correlated with increased expression of the intestinal fatty acid-binding protein, LPS-binding protein, and sCD14 in circulation, all of which are considered biomarkers of leaky gut [170], highlighting the potential link between aging, inflammation, and increased intestinal permeability.

## Altered intestinal permeability in ageing

Though several animal studies in mice, rats, and baboons have demonstrated age-related impacts on intestinal permeability [71, 98, 159], a handful of studies conducted in humans have yielded contradictory results [99, 141, 163]. In a cross-sectional study of 85 elderly individuals (60–82 years) and 130 younger adults (19–59 years), no difference in small intestinal permeability index (percentage recovery of lactulose/percentage recovery of mannitol) was found when comparing elderly and young populations in the absence of disease [163].

By contrast, a follow-up study investigating 82 ileal biopsies from young (7–12 years), adult (20–40 years), and aged (67–77 years) individuals for inflammatory cytokines, barrier integrity, and cytokine production in response to microbial challenges demonstrated an upregulated production of IL-6 which was accompanied by reduced ex vivo transepithelial resistance [99].

A more recent study assessing intestinal permeability in vivo by the multi-sugar test (1 g sucrose, 1 g lactulose, 0.5 g L-rhamnose, 1 g sucralose, and 1 g erythritol) in 48 elderly and 52 young adults found that while gastroduodenal permeability was lower in elderly vs. young adults, lactulose/mannitol ratio, reflecting small intestinal permeability, sucralose/erythritol ratio, reflecting colonic permeability, and whole gut permeability was unchanged between healthy elderly and healthy young adults [179]. Similarly, no significant difference in transepithelial resistance and ex vivo intestinal permeability of the colon was found between elderly and young adults [179].

A reason for the discrepancy in results may be the presence or absence of GI symptoms among individuals assessed. A recent study investing intestinal permeability in aged populations with and without self-reported GI symptoms found that older adults with GI symptoms displayed significantly higher levels of zonulin in plasma compared to older adults representing the general elderly population and senior orienteering athletes (model of healthy aging) [59].

Given this growing evidence for altered intestinal barrier morphology and function in the aging population and the growing evidence for the link between leaky gut and systemic inflammation [82, 156], it is highly plausible that intestinal barrier dysfunction and subsequent systemic inflammation could contribute to the onset of age-related diseases.

## Link between chronic gi inflammatory disorders and age-related disease

Several age-related conditions, such as OA, COPD, CVD, T2DSeveral age-related conditions, such as OA, COPD, CVD, T2D, and cognitive decline associated with dementia have been linked with gut dysbiosis and interestingly are associated with increased risk among IBS and IBD patients [64, 79, 136, 150, 192]. Given the well-established GI phenotype in both IBD and IBS, and their common leaky gut pathology, probing the relationship between GI inflammatory disorders and common age-related conditions may provide insight into a potential relationship between the intestinal barrier and inflammation (Table 3).

**Table 3:** Common age-related diseases and their relationship to IBS, IBD, and dysbiosis

	Risk in IBS and/or IBD	Evidence of dysbiosis
Osteoarthritis	Patients with IBS have a two-fold increased risk of OP (Wongtrakul et al. [180]) Patients with IBD have a 32% increased risk of developing OP (Hidalgo et al. [70])	*↑Clostridium *sensu stricto, *Bacteroides*, *Intestinibacter*, and *Limosilactobacillus* *↓Collinsella*, *Megasphaera*, *Agathobaculum*, *Mediterraneibacter*, *Clostridium XIV*, and *Dorea *(Akinsuyi and Roesch [4])
Chronic obstructive pulmonary disease	UC and CD patients have an increased risk of asthma and bronchitis (Bernstein et al. [14]) IBD patients of 46% higher rate of bronchiectasis, a 52% higher rate of pulmonary vasculitis and interstitial pneumonia, a 35% higher risk for lung nodules, a 16% higher rate of pulmonary fibrosis, and a 5.5% higher rate of asthma than non-IBD patients (Pemmasani et al. [123])	*↑Bifidobacteriaceae*, *Eubacteriaceae*, *Lactobacillaceae*, *Micrococcaceae*, *Streptococcaceae*, and *Veillonellaceae* *↓Desulfovibrionaceae*, *Gastranaerophilaceae*, *Selenomonadaceae*, *Bacilli*, and *Clostridia *(Bowerman et al. [20])
Cardiovascular disease	IBD is associated with a 21% higher risk of CVA and an 18% increased risk of IHD (Singh et al., 146) IBD patients have an increased risk of stroke (HR = 1.29) (Xiao et al. [183]) RR of CVA was 1.25 in IBD patients compared to non-IBD patients. RR of CHD was 1.17 for IBD patients compared to non-IBD patients (Sun and Tian [154]) Patients with IBD are associated with a 24% risk of IHD (Feng et al. [49])	*↑Enterobacteriaceae*, *Lactobacillus*, and *Streptococcus taxa* in CAD patients *↓Bacteroidetes* and *Lachnospiraceae* in CAD patients (Choroszy et al. [30]) *↑Catabacter*, *Robinsoleilla*, *Serratia, Enterobacteriaceae*, *Ruminococcus torques*, *Parasutterella*, *Escherichia*, *Shigella*, and *Klebsiella* in HT patients *↓Sporobacter*, *Roseburia hominis*, *Romboutsia* spp., and *Roseburia* in HT patients (Naik et al. [114])
Type 2 diabetes	CD patients have an increased risk of T2D (HR = 1.29), whilst CD patients do not (Dregan et al. [44]) UC and CD patients have an increased risk of T2D (SIR: 1.54 and 1.57, respectively) (Jess et al. [77])	*↑Actinobacteria*, *Firmicutes* in T2DM *↓Bacteroidetes* in T2DM (Que et al. [131])
Dementia	IBD patients exhibit deficits in attention, executive function, and working memory when compared to healthy controls (Hopkins et al. [72]) Risk of dementia in IBD patients is significantly higher than in the general population (RR = 1.35) (Zhang et al. [190]) Risk of developing dementia significantly increased after IBD diagnosis (HR = 1.27) (Liu et al. [97])	*↓Faecalibacterium* in patients with SCD compared with normal controls (Sheng et al. [142]) *↑Proteobacteria*, *Bifidobacterium*, and *Phascolarctobacterium* in AD *↓Firmicutes*, *Clostridiaceae*, *Lachnospiraceae*, and *Rikenellaceae* in AD (Hung et al. [75]) *↑ Eggerthellaceae*, *Desulfovibrionaceae*, *Coriobacteriaceae*, and *Anaerovoracaceae* in DLB *↓Ruminococcaceae* in DLB (Nishiwaki et al. [117])

*AD*, Alzheimer’s disease; *CD*, Crohn’s disease; *CAD*, coronary artery disease; *CHD*, coronary heart disease; *CVA*, cerebrovascular accidents; *DLB*, dementia with Lewy body; *HR*, hazard ratio; *HT*, hypertensive; *IBD*, inflammatory bowel disease; *IHD*, ischemic heart disease; *OP*, osteoporosis; *RR*, risk ration; *SIR*, standardized incidence ratios; *SCD*, subjective cognitive decline; *T2D*, type-2 diabetes; *UC*, ulcerative colitis

### Osteoporosis

Much of the research conducted to investigate the connection between gut and bone health has primarily focused on fecal microbiota. Mounting evidence has linked changes in the gut microbiota to the pathophysiology of osteoporosis. A recent meta-analysis combining and re-examining five publicly available 16S rRNA partial sequence data sets to identify gut bacteria, found consistent microbiome population changes associated with osteoporosis across different cohorts. A significant shift in the microbial composition of osteoporotic patients, driven by an increase in the relative abundance of *Clostridium *sensu stricto, *Bacteroides*, *Intestinibacter*, and *Limosilactobacillus*, was seen alongside depletion of members of the genera *Collinsella*, *Megasphaera*, *Agathobaculum*, *Mediterraneibacter*, *Clostridium XIV*, and *Dorea* [4]. While it remains debated whether microbial changes directly contribute to bone loss or are a secondary consequence of systemic inflammation, current evidence points to a potentially causal link [172]. Growing evidence indicates that microbiota changes may influence bone metabolism, potentially through inflammatory mechanisms or altered calcium and vitamin D absorption. However, it is also possible that in conditions like postmenopausal osteoporosis, systemic inflammation alters the microbiota, making it unclear whether these microbiota changes are primarily causal or secondary. Severely, recent reviews have explored this burgeoning field of osteomicrobiology [16, 74, 80].

To our knowledge, no studies have been undertaken to investigate intestinal morphology, inflammation, or permeability in osteoporosis; however, an association between IBS, IBD, and osteoporosis exists.

A recent systematic review and meta-analysis involving five studies found a significantly increased risk of osteoporosis among IBS patients [180]. The pooled analysis from nearly 530,000 individuals found an approximately two-fold increased risk of osteoporosis among IBS sufferers. A significant association between IBD and the risk of developing osteoporotic fractures has also been noted [70]. A meta-analysis of seven studies concluded that there is a 32% increased risk of osteoporosis among IBD patients [70].

### Chronic obstructive pulmonary disease

There is emerging evidence suggesting a connection between intestinal barrier dysfunction and COPD. Small intestinal permeability during acute exacerbations of COPD has been shown to be significantly increased [150]. Compared to stable conditions, the urinary lactulose/rhamnose ratio, reflecting small intestine permeability, was significantly increased in exacerbated COPD patients, while urinary S/E and Su/R ratio, reflecting proximal colon and gastric/duodenal permeability, respectively, was unchanged when comparing exacerbated vs. stable condition COPD [150]. In rodent models of COPD, intestinal morphology studies have also indicated swollen intestines with darkened and grey mucosa, neutrophil infiltration, and regional epithelial shedding alongside reduced expression of occludin and ZO‑1 [184].

Strong evidence indicates that intestinal microbiota is altered in patients with COPD. A recent study found a significant difference in overall community composition between COPD and healthy gut microbiomes, without a significant difference in diversity [20]. Specific genera increased in abundance in COPD were *Streptococcus*, *Rothia*, *Romboutsia*, *Intestinibacter*, and *Escherichia*. While genera decreased in COPD included *Bacteroides*, *Roseburia*, and *Lachnospira* as well as several unnamed genera of *Ruminococcaceae* [20].

Emerging research also shows that intestinal microbiota may actually play a role in the onset and progression of COPD, with a recent mendelian randomization study demonstrating a causal relationship exists between certain gut microbiota (*Desulfovibrionales*, family Desulfovibrionaceae, family *Peptococcaceae*, family *Victivallaceae*, and genus *Marvinbryantia*) and COPD [176].

There is extensive literature documenting pulmonary disease in IBD [169]. Approximately 40–60% of IBD patients have some degree of subclinical lung involvement evidenced through alterations in pulmonary function testing [69, 78, 108]. An early matched-cohort study with 8072 IBD patients and 31,365 patients without disease found that both UC and CD patients had a significantly greater likelihood of having asthma and bronchitis compared to healthy controls [14]. While a large retrospective observational study including 175,012 IBD and non-IBD patients which assessed the prevalence of various diseases found that patients with IBD had a 46% higher rate of bronchiectasis, 52% higher rate of pulmonary vasculitis and interstitial pneumonia, 35% higher risk for lung nodules, 16% higher rate of pulmonary fibrosis, and a 5.5% higher rate of asthma than non-IBD patients [123].

In line with this, the risk of new-onset IBD was shown to be higher in populations with COPD compared to the general population [21, 90]. The incidence of CD and UC was 55% and 30%, respectively, higher in patients with COPD in one study [21] and yielded risk ratios of 2.29 for UC and 1.79 for CD in another [90]. Most importantly, IBD has been highlighted as a risk factor for increased mortality in patients with pre-existing COPD or asthma-COPD [168], whereas a meta-analysis of population-based studies showed that CD was associated with an increased risk of death by COPD (mortality ratios = 2.55) [46].

### Cardiovascular disease

CVD is an umbrella term that describes a disease of the heart or blood vessels, and as such includes a collection of conditions including coronary artery disease, high blood pressure, congestive heart failure, cardiac arrest, and stroke as well as various others. In both sexes, the risk of CVD increased markedly with age and some evidence points towards increased risk of CVD among IBD sufferers.

One meta-analysis published in 2014, which analyzed data from nine studies (2424 cerebrovascular accidents across five studies and 6478 ischemic heart disease events in six studies), found that IBD is associated with an 18% increase in the risk of cardiovascular morbidity particularly in women [146]. A more recent study meta-analysis including 27 articles, of which 11 studies reported the risk of CVD incidence and 16 studies reported the risk of cardiovascular disease death also found a positive association between IBD and higher risk of CVD incidence, particularly in females [154]. Similarly, a meta-analysis conducted with 10 cohort studies found patients with IBD were associated with a 24% increased risk of ischemic heart disease [49]. A link between IBD and stroke has also been noted, with a 2015 meta-analysis showing that patients with IBD experienced a modest increase in risk for the development of stroke compared with non-IBD patients [183].

The intestinal microbiome is also known to play a role in age-related cardiovascular inflammation. Some studies have shown the presence of bacterial DNA within atherosclerotic plaques; in addition, microbial changes have been noticed in CVD patients. A recent meta-analysis of seven papers focused on coronary artery disease found that alpha-diversity was significantly decreased [30]. The most consistent results across studies highlighted a reduced abundance of *Bacteroidetes* and *Lachnospiraceae* in patients with coronary artery disease, and an increased abundance of *Enterobacteriaceae*, *Lactobacillus*, and *Streptococcus taxa* [30].

A systematic review of eight papers published in 2022 also concluded that overall the abundance of *Catabacter*, *Robinsoleilla*, *Serratia*, *Enterobacteriaceae*, *Ruminococcus torques*, *Parasutterella*, *Escherichia*, *Shigella*, and *Klebsiella* was increased in hypertensive patients while a decreased abundance of *Sporobacter*, *Roseburia hominis*, *Romboutsia* spp., and *Roseburia* was often seen [114].

With regard to intestinal barrier function, increased intestinal permeability has been reported in multiple human and animal studies of CVD [94]. Dysfunctional mucosal barrier [5] and increased small and large intestine permeability were observed in early investigations of chronic heart failure establishing a role for intestinal barrier function specifically [139]. In patients with chronic heart failure muscle thickness in the terminal ileum and colon was substantially increased, as was small intestinal permeability (lactulose/mannitol ratio) and large intestinal permeability (sucralose excretion) when compared to controls [139]. Higher concentrations of adherent bacteria were also found within the mucus of chronic heart failure patients compared with control subjects [139].

### Type 2 diabetes

Advanced age is an important independent risk factor for type 2 diabetes (T2D), and a substantial body of literature describes the role of the gut microbiome in its pathophysiology [89].

Reports vary with respect to the association between T2D and specific taxonomic groups, a recent study by Gurung and colleagues reviewed 42 human studies on the topic and identified microbial trends associated with the disease [65]. Commonly reported findings included a negative association between T2D and the genera of *Bifidobacterium*, *Bacteroides*, *Faecalibacterium*, *Akkermansia*, and *Roseburia* and a positive association with *Ruminococcus*, *Fusobacterium*, and *Blautia* [65]. A recent study comparing gut microbiota from a cohort of healthy and diabetic Chinese individuals revealed a significant decrease in overall gut microbiota diversity and butyrate-producing bacteria, such as *Bifidobacterium* and *Akkermansi*a in diabetic patients compared with healthy controls [95]. A systematic review and meta-analysis of seven studies investigating microbial dysbiosis in T2D (600 T2D cases, 543 controls, 1143 samples in total) found significant differences in beta diversity, but not alpha diversity, between individuals with T2D and controls [131]. Taxonomic abundances of bacterial phyla grouped by individual study showed consistent trends: increased relative abundances of *Firmicutes* (class *Negativicutes*, order *Selenomonadales*, family *Veillonellaceae*) and *Actinobacteria* (class *Actinobacteria*) and decreased relative abundances of *Bacteroidete*s (class *Bacteroidia* or order *Bacteroidales*) in patients with T2D [131].

Several studies have investigated intestinal barrier dysfunction in T2D [35, 76, 191]. Overall, intestinal barrier function has been shown to be impaired in T2D patients, indicated by increased serum levels of LPS-binding protein, I-FABP, LPS, zonulin, and ZO-1 [35, 76, 188, 191]. With regard to intestinal morphology, these authors could not find any research relating to the human condition,however, studies conducted in rodent models consistently show altered ileal and colonic crypt morphology [125, 171].

Few studies have examined the relationship between inflammatory GI conditions and T2D. A matched cohort study in the UK involving 12 203 UC and 7628 CD patients showed a significantly increased risk of T2D in patients with UC but not in patients with CD [44]. A nationwide population-based cohort of 6,028,844 subjects in Denmark showed that both UC and CD were associated with significantly increased risk of T2D, with the risk highest in the first year after a diagnosis of IBD, but remaining increased for 20 or more years following the diagnosis [77].

### Cognitive decline associated with dementia

A significant connection between gut microbiota and brain function exists [39, 68]. Although age is the predominant risk factor for dementia, few studies have investigated true age-related cognitive decline (in the absence of disease). Most studies to date have focused on dementia, which refers to a collection of diseases including Alzheimer’s disease, vascular dementia, Lewy body disease, and frontotemporal dementia, all of which affect memory, thinking, and the ability to perform daily activities. Recently, a link between microbial dysbiosis and dementia has been identified.

In a recent study, the abundance of phylum *Firmicutes*, class *Clostridia*, order *Clostridiales*, family *Ruminococcaceae*, and genus *Faecalibacterium* in fecal samples all showed a trend towards a progressive decline when comparing normal control patients with individuals with subjective cognitive decline (SCD), the earliest symptomatic manifestation of preclinical Alzheimer’s disease, and cognitive impairment [142]. Specifically, the abundance of *Faecalibacterium* was significantly decreased in patients with SCD compared with normal controls [142].

A 2022 meta-analysis consisting of 378 healthy controls and 427 patients with Alzheimer’s disease concluded that patients with Alzheimer’s disease had significantly reduced microbial diversity as compared to healthy controls. Taxonomic abundance was also altered, with an increased abundance of *Proteobacteria*, *Bifidobacterium*, and *Phascolarctobacterium*, alongside a reduced abundance of *Firmicutes*, *Clostridiaceae*, *Lachnospiraceae*, and *Rikenellaceae* [75]. Similarly, recent work comparing fecal microbiota in 28 dementia with Lewy body (DLB) patients and 147 controls found that four families were increased (*Eggerthellaceae*, *Desulfovibrionaceae*, *Coriobacteriaceae*, and *Anaerovoracaceae*) and one family was decreased (*Ruminococcaceae*) in DLB patients when compared to controls after adjusting for the confounding factors. While at the genus level, three genera were increased (*Collinsella*, *Eggerthella*, and *Ruminococcus torques*) and seven genera were decreased (*Agathobacter*, *Lachnospiraceae* ND3007 group, *Butyricicoccus*, *Coprococcus*, *Faecalibacterium*, *Fusicatenibacter*, and *Haemophilus*) in DLB patients compared to controls [117].

Over the past several decades, substantial research has been undertaken to better understand the role of the intestinal barrier in neurological decline [121, 122, 135, 152]. Aside from differences in beta diversity and changes in taxonomic composition, cognitive decline and dementia have been associated with increased markers of intestinal permeability such as serum diamine oxidase, d-lactic acid, and endotoxin [121, 152]. Changes in intestinal morphology and tight junction expression have also been noted in several neurodegenerative conditions in humans and rodents alike [32, 52, 119, 124, 173]. The connection between intestinal disorders/intestinal barrier dysfunction and neurodegenerative diseases has been extensively reviewed elsewhere [54, 122, 157, 182].

Reports of cognitive impairment in IBD have been mixed. While several studies have provided evidence for a significant reduction in verbal IQ among patients with IBD and IBS compared to healthy controls [8, 41], subsequent studies found no evidence of major verbal memory or cognitive deficits among IBD patients [25]. A recent systematic review and meta-analysis including 11 studies found that people with IBD showed significant deficits in attention, executive function, and working memory when compared to healthy controls [72]. Furthermore, several recent population-based studies and meta-analyses have found a unidirectional association between IBD and dementia, with the overall risk of dementia in IBD patients significantly higher than that of the general population [97, 133, 190].

## Conclusion

Chronic low-grade systemic inflammation is an important factor associated with age-related disease burden. A growing body of literature supports the notion that the gut may contribute to the inflammatory burden in aging and thus contribute to the development of age-related diseases. While there is emerging evidence of a potential leaky gut phenotype in several age-related diseases, care must be taken when assessing and interpreting the connection between hyperpermeability and various disease states, given that it is not yet established whether increased permeability is in fact deleterious. Given that the gut harbors a large proportion of the body’s lymphocyte population, its role in the development and progression of age-related chronic systemic inflammation warrants further investigation to determine whether altered intestinal permeability, intestinal inflammation, and morphological changes in the gut are a cause or consequence of diseases associated with aging.

## Abbreviations

AD: Alzheimer’s disease
CVD: Cardiovascular disease
ChAT: Choline acetyltransferase
COPD: Chronic obstructive pulmonary disease
CVA: Cerebrovascular accidents
CAD: Coronary artery disease
CHD: Heart disease
CRP: C-reactive protein
CD: Crohn’s disease
DLB: Dementia with Lewy body
GI: Gastrointestinal
HR: Hazard ratio
HT: Hypertensive
IBD: Inflammatory bowel disease
IBS: Irritable bowel syndrome
IHD: Ischemic heart disease
LPS: Lipopolysaccharide
NADPH: Nicotinamide adenine dinucleotide phosphate
NF: Nuclear factor
nNOS: Neuronal nitric oxide synthase
OA: Osteoarthritis
RR: Risk ration
SIR: Standardized incidence ratios
SCD: Subjective cognitive decline
ROS: Reactive oxygen species
TJ: Tight junction
TNF-α: Tumor necrosis factor-alpha
TGFβ: Transforming growth factor-β
T2D: Type-2 diabetes
UC: Ulcerative colitis
